# The impact of physical exercise on academic satisfaction among middle-school students: the mediating roles of social support and depression

**DOI:** 10.3389/fpsyg.2025.1653610

**Published:** 2026-01-06

**Authors:** Xing Huo, Jinhuan Guan, Haibo Tian

**Affiliations:** Department of Physical Education, Shaoxing University, Shaoxing, China

**Keywords:** physical exercise, academic satisfaction, social support, depression, middle-school

## Abstract

**Introduction:**

The association between physical exercise and academic satisfaction is a crucial topic in recent research. However, existing literature has paid insufficient attention to exploring the influence of physical exercise on academic satisfaction, particularly regarding the mediating role of social support and depression.

**Methods:**

A sample of 564 Chinese middle-school students was randomly recruited from five high schools in Shaoxing City. We proposed a hypothesized model to examine the relationship between physical exercise, academic satisfaction, social support, and depression.

**Results:**

Results from PROCESS v4.1 for SPSS revealed that physical exercise had a significant and positive impact on both academic satisfaction and social support; Social support showed a positive association with academic satisfaction; Physical exercise, social support, and academic satisfaction were significantly negatively correlated with depression; Furthermore, social support and depression partially mediated the relationship between physical exercise and academic satisfaction.

**Discussion:**

These findings provide empirical evidence into elucidating how physical exercise promotes high school students’ academic satisfaction. The limitations and future directions of these findings are discussed.

## 1 Introduction

Mental health issues among middle school students have garnered extensive attention due to their heightened susceptibility to conflicts arising from parental or peer relationships, as well as emotional disorders (Arnett, 1999; Romeo et al., 2017). Recent research has revealed that middle-school students often experience significant academic pressure, which can leads to various mental health problems such as depression and anxiety (Pascoe et al., 2020). In recent years, the phenomenon of “involution,” driven by academic pressure, has become increasingly prominent among Chinese teenagers. Cultivating appropriate academic emotions can help enhance their academic performance (Dong and Yu, 2010). Therefore, it is imperative not to delay efforts aimed at enhancing the relationship between mental health and academic satisfaction for middle school students.

Physical exercise confers benefits on individuals’ physical health, while also exerting a positive influence on their mental well-being and academic performance (Curran et al., 2020). Academic satisfaction, which refers to students’ contentment with their academic achievements and overall school life (Geng, 2016), is considered a fundamental aspect and pivotal factor affecting mental health (Yu, 2022) as well as life satisfaction (Bücker et al., 2018). Previous literature provides supporting evidence for the impact of physical exercise on academic satisfaction (Li and Zhang, 2022; Singh et al., 2012). For instance, middle school students engaging in moderate or low-intensity exercise reported higher levels of subjective well-being and academic satisfaction compared to those participating in high-intensity physical activities (Chen, 2004). Given the escalating academic pressure faced by middle school students nowadays, it becomes increasingly urgent to investigate the influence of physical exercise on their academic satisfaction.

Previous research has confirmed the direct impact of physical exercise on individuals’ academic satisfaction (Gaudreau et al., 2015). However, limited attention has been given to the complexity and indirect nature of this relationship. In other words, there may exist some mediating variables that influence the role of physical exercise in determining an individual’s academic satisfaction. Ryu et al. (2021) discovered a partial mediating effect of aerobic fitness between physical activity and academic satisfaction. Additionally, studies have indicated that students’ cognition (Rasberry et al., 2011) and self-esteem (Kayani et al., 2018) play significant roles in the relationship between physical exercise and academic satisfaction. Recent research suggests that social support and depression can also significantly affect academic satisfaction (Helgeson, 2003; Schuch et al., 2016), yet these factors have not been considered as potential mediators when examining the impact of physical exercise on academic satisfaction. After the implementation of the “double reduction” policy in China, the role of physical education in the growth of teenagers has undergone significant changes, including the status of the courses and the degree of attention from parents. Therefore, it is essential to construct a hypothetical model to explore the relationship between physical exercise and academic satisfaction by incorporating social support and depression as mediating variables.

## 2 Literature review and development of hypotheses

### 2.1 Physical exercise and academic satisfaction

Academic satisfaction has been extensively utilized in the fields of education, psychology, and other disciplinesto assess students’ satisfaction with their educational experiences (Richardson et al., 2012; Zajacova et al., 2005). Academic satisfaction refers to students’ overall satisfaction with their academic performance and school life (Geng, 2016). It encompasses three primary dimensions: learning satisfaction, hardware satisfaction and teaching satisfaction (Li and Wang, 2009; Zhang et al., 2021). In this study, academic satisfaction primarily reflects middle school students’ satisfaction with school teaching, environmental resources, and personal learning progress—a discrepancy between desired outcomes and actual achievements. Physical exercise serves as a principal approach for enhancing physical and mental well-being while alleviating psychological stressors (Ma et al., 2023) and fostering positive emotions. Physical exercise refers to regular planned physical activities (Caspersen et al., 1985), encompassing three key aspects: intensity, frequency, and duration (Liang, 1994). This study posits that physical exercise constitutes a routine physical activity undertaken by middle school students to enhance their physical fitness levels and promote overall health.

Previous studies have provided indirect and reliable evidence to examine the impact of physical activity on academic satisfaction. Taking adolescents as an example, Moral-García et al. (2019) investigated the association between physical activity and satisfaction with physical education, revealing that students with higher levels of physical exercise exhibit greater physical education satisfaction. Furthermore, a study by Moral-Garcia et al. (2021) demonstrated that regular physical exercise enhances school satisfaction among adolescents. Recent research has also confirmed that academic satisfaction is considered a crucial component of overall life satisfaction for this population, and engaging in physical exercise promotes both academic and life satisfaction (Mazur et al., 2018; Strózik et al., 2016). Additionally, demographic variables such as grade level, gender, and age significantly influence the relationship between physical exercise and academic satisfaction among middle school students (Dong, 2017). Based on these findings, it can be inferred that physical exercise may exert a positive impact on the academic satisfaction of middle school students.

### 2.2 Social support and its mediating role

The term “social support” refers to the perception of care and assistance from family members, friends, and society (Heaney and Israel, 2008; Tay et al., 2013). It serves as a connection between these sources and is operationalized through a social support network (Cohen, 1988). Social support encompasses three dimensions: subjective support, objective support, and support utilization (Xiao, 1994). It is commonly used to elucidate the impact of societal assistance on an individual’s mental well-being and quality of life, including emotional support, practical aid, information provision, and advice (Tamers et al., 2011). Gallagher and Vella-Brodrick (2008) discovered that social support fosters positive emotions by influencing affective states and constructive behaviors in teenagers. Consequently, it enhances their academic performance and satisfaction levels. Furthermore, adolescents with higher levels of social support also exhibit greater academic satisfaction (Jia and Cheng, 2024). Additionally, physical exercise represents an effective means to enhance individual social support. As exercise motivation increases among adolescents, they become more inclined to engage in sports activities while concurrently obtaining stronger social support (Downs and Hausenblas, 2005). This study highlights that improvements in youth sports autonomy (Hagger et al., 2003) and the emergence of challenging athletic pursuits (Martínez-Martí and Ruch, 2016) compel them to seek increased familial and educational assistance.

The literature suggests that physical activity can improve quality of life and satisfaction by increasing friendships and supportive recognition from others (Kvam et al., 2016; Pazzaglia et al., 2020). Individuals who participate in physical exercise have higher perceived social support and higher life satisfaction (Chen et al., 2021b). Lai and Ma (2016) noted that social support from family and friend co-moderates satisfaction and that appropriate physical activity can promote the improvement of social support by preventing health risks. For adolescents, participation in physical activity increases social support, enhances self-esteem and improves body image, resulting in positive emotions and high levels of satisfaction (Babiss and Gangwisch, 2009). In other words, physical exercise can enable students to obtain greater social support, social support can enhance students’ academic satisfaction, and social support may indirectly promote the influence of physical exercise on academic satisfaction.

The existing literature suggests that engagement in physical activity can enhance quality of life and satisfaction through the facilitation of friendships and supportive recognition from others (Kvam et al., 2016; Pazzaglia et al., 2020). Individuals who actively participate in physical exercise tend to perceive higher levels of social support and experience greater life satisfaction (Chen et al., 2021a). Lai and Ma (2016) observed that family and friend-based social support co-moderates satisfaction, while appropriate physical activity can foster improved social support by mitigating health risks. For adolescents, involvement in physical activity not only boosts social support but also enhances self-esteem, improves body image, generates positive emotions, and fosters high levels of satisfaction (Babiss and Gangwisch, 2009). In essence, engaging in physical exercise enables students to acquire increased social support which subsequently bolsters academic satisfaction indirectly.

### 2.3 Depression and its mediating role

Depression is a negative mental condition characterized by persistent feelings of sadness, loss of interest and pleasure, fatigue, and lack of energy (Roepke and Seligman, 2016). Middle school students often encounter academic pressure, social expectations, self-imposed demands, and uncertainty about the future, making them more susceptible to depression which significantly impacts their academic performance as well as their physical and mental well-being (Chen et al., 2021a; Yu et al., 2023). There appears to be a robust association between physical exercise and depression (Marques et al., 2020; Schuch et al., 2016). Mammen and Faulkner (2013) reported that physical exercise has a moderate antidepressant effect and even a minimal amount of physical activity can contribute to preventing depression. Kim et al. (2019) also confirmed that engaging in appropriate physical activity is beneficial for reducing depressive symptoms. Furthermore, depression seems to be one of the primary factors contributing to decreased academic satisfaction levels among individuals (Kayani et al., 2018). According to the quality-stress theory proposed by Broerman (2018), stressors play a significant role in the development of depression since they transform potential depressive tendencies into actual experiences through adverse life events. Schütz et al. (2013) found that experiencing positive emotions can effectively inhibit depressive emotions leading to improved academic performance and satisfaction levels. Pekrun et al. (2002) also supported this perspective by demonstrating that students who experience negative emotions such as depression or anxiety tend to have lower levels of academic satisfaction.

It is imperative to attend to the intricate relationship between physical exercise, academic satisfaction, and depression (Moksnes et al., 2016). Empirical evidence suggests that physically active students exhibit lower levels of depression and stress, as well as higher levels of happiness (Moljord et al., 2011). Across different countries, active engagement in physical activity has been linked to increased life satisfaction among adolescents (Grant et al., 2009). Simultaneously, higher levels of physical activity have been associated with reduced depression rates and enhanced academic performance and satisfaction (Dorfman, 2015). Additionally, Kayani et al. (2018) confirmed the mediating role of depression in the relationship between physical exercise and academic satisfaction. Furthermore, regular participation in physical exercise has demonstrated improvements in overall quality of life and contentment (Warburton et al., 2006). Consequently, both physical exercise and academic satisfaction exert a positive influence on alleviating depression; conversely, depression significantly impacts the enhancement of academic satisfaction. Depression may serve as a mediator in the association between physical exercise and academic satisfaction.

### 2.4 The hypotheses of this study

In summary, the objective of this study is to investigate the associations among physical exercise, academic satisfaction, social support, and depression among Chinese middle-school students. Based on the aforementioned information, we formulated the following hypotheses:

*H1*: Physical exercise has a significant positive impact on academic satisfaction.

*H2*: Physical exercise has a significant positive effect on social support.

*H3*: Physical exercise has a significant negative effect on depression.

*H4*: Social support has a significant positive impact on academic satisfaction.

*H5*: Social support has a significant negative effect on depression.

*H6*: Depression has a significant negative impact on academic satisfaction.

*H7*: Social support mediates the relationship between physical exercise and academic satisfaction.

*H8*: Depression mediates the relationship between physical exercise and academic satisfaction.

## 3 Methods

### 3.1 Measurements

The Physical Activity Rating Scale-3 (PARS-3), initially developed by Hashimoto (1990) and subsequently revised by Liang (1994), was employed to assess the levels of physical exercise among middle school students. It comprised three items encompassing three dimensions: intensity (1 item), frequency (1 item), and duration (1 item). The Likert 5-point scoring method was utilized for quantifying the items, while the total score was obtained by multiplying the scores of these three items together; higher scores indicated greater levels of physical exercise among participants. Regarding exercise intensity options, “1” denoted light exercise such as walking, whereas “5” represented intense and sustained exercises like swimming. Extensive research conducted on Chinese adolescent populations has consistently demonstrated that this scale possesses high reliability and validity. In a recent study involving adolescents, this scale exhibited robust psychometric properties (Liu et al., 2024). In our present investigation, PARS-3 yielded a Cronbach’s coefficient of 0.833.

The Satisfaction with Academic Scale (SWAS), developed by Li and Wang (2009), was employed to assess the extent to which students positively evaluate the overall quality of their academic experience. It comprises three dimensions encompassing a total of 12 items: learning satisfaction (4 items), hardware satisfaction (4 items), and teaching satisfaction (4 items). A Likert 5-point scoring method was utilized for item quantification, where “1” indicates a complete lack of conformity and “5” represents complete conformity. Higher scores indicate greater levels of academic satisfaction. The statement regarding hardware satisfaction was formulated as follows: “I am dissatisfied with the school’s management system.” Ji (2018) confirmed that SWAS demonstrated good reliability and validity among middle school students. In this study, Cronbach’s *α* coefficients for scale dimensions were found to be 0.862, 0.736, and 0.940.

The Social Support Rate Scale (SSRS), developed by Xiao (1994) and revised by Ye and Dai (2008), was employed to assess the levels of social support among middle school students. It consists of 17 items that encompass three dimensions: subjective support (5 items), objective support (6 items), and support utilization (6 items). The SSRS utilized a 5-point Likert scale ranging from “1” denoting unacceptable to “5” representing acceptable. Higher total scores indicate greater levels of social support. Regarding subjective support, participants were asked to rate the statement: “Most of my classmates showed significant concern for me.” This scale has demonstrated notable efficacy in adolescent populations as evidenced by recent research conducted by Wang and Wu (2022). In our current study, the internal consistency reliability coefficient (Cronbach’s α) for the SSRS was calculated at an impressive value of 0.965.

The Self-Rating Depression Scale (SDS), developed by Zung (1965), is utilized for assessing symptoms associated with depressive states, their severity, and changes. SDS consists of 20 declarative sentences and corresponding question entries, with 10 reverse-scored questions such as “I feel hopeful about the future.” Ratings on the SDS are based on a 4-point scale where “1” indicates never or occasionally while “5” represents always; Higher scores indicate greater levels of depression. The scale has been widely used in China and has high reliability in studying adolescent depression (Li, 2013). In the current study, the Cronbach’s coefficient of the SDS was 0.794.

According to the existing literature (Shi et al., 2023; Song et al., 2010; Yao et al., 2023), 8 demographic variables were introduced as control variables in the proposed model. These variables include gender, grade, origin of student, religious belief, family economic condition, parents’ education background, only child status, and accommodation situation.

### 3.2 Procedures and data analysis

The data for the study were collected in December 2023 from five prominent secondary schools located in Shaoxing, a Jiangnan Water Town in Southeast China. Among them, three are senior high schools, two are key schools, and two are rural high schools. In this study, the questionnaire was distributed randomly (i.e., every 5th person) among students at these selected schools with prior consent obtained from school authorities. All participants willingly took part in the study after obtaining written informed consent from their parents or guardians. Prior to completing the survey, participants were provided with an explanation of key concepts (e.g., academic satisfaction) related to the outcomes to ensure clarity and understanding. Any queries raised by participants were promptly addressed and resolved to prevent any potential misunderstandings. A total of 574 questionnaires were collected through field distribution; however, ten incomplete questionnaires with repetitive responses were excluded from analysis. Finally, all hypotheses were tested using 564 valid questionnaires.

SPSS 27.0 was utilized for data analysis in this study. Firstly, descriptive statistics were employed to examine the basic information of the sample, while frequency analysis was conducted to analyze the composition and characteristics of the population. Secondly, Pearson’s correlation coefficient was used to evaluate the correlation among all variables. Finally, PROCESS v4.1 for SPSS 27.0 was subsequently applied to construct a structural equation model for hypothesis validation in this study.

## 4 Results

### 4.1 The information of demographic variables

As indicated in Table 1, accounting for 57.1% of the total participants were female students. The majority of the participants were junior high school (61.9%), with a significant proportion having no religious belief (83.3%) and being non-only children (62.8%). Urban residents constituted 64.4% of the student population, while the majority were not resident students (87.9%). Furthermore, a considerable percentage of participants’ parents possessed education background of college or undergraduate (41.8%), and their families economic condition enjoyed a moderately rich status (70.4%).

**Table 1:** Respondent profile (*n* = 564).

Characteristics	Frequency (*n*)	Percentage (%)
Gender		
Male	242	42.9
Female	322	57.1
Grade		
Junior high school	349	61.9
Senior high school	215	38.1
Religious belief		
Yes	94	16.7
No	470	83.3
Only child		
Yes	210	37.2
No	354	62.8
Origin of student		
Countryside	201	35.6
Urban	363	64.4
Resident student		
Yes	68	12.1
No	496	87.9
Parents education background		
Primary school	9	1.6
Junior school	105	18.6
Education technical secondary school or above	154	27.3
College or undergraduate	236	41.8
Postgraduate and above	60	10.6
Family economic condition		
Great rich	22	3.9
Relatively rich	37	6.6
Moderately rich	397	70.4
Not very rich	91	16.1
Not rich at all	17	3.0

### 4.2 Descriptive statistics among the variables

Descriptive statistics and correlation analysis were performed on the core variables in this study, and the means, standard deviations and correlation coefficient are presented. As shown in Table 2, Pearson’s correlation analysis revealed a significant positive association between physical exercise and academic satisfaction (*r* = 0.222, *p* < 0.01), as well as social support (*r* = 0.160, *p* < 0.01). Additionally, a significant negative correlation was observed between physical exercise and depression (*r* = −0.133, *p* < 0.01). Academic satisfaction exhibited a substantial positive correlation with social support (*r* = 0.683, *p* < 0.01). Furthermore, both academic satisfaction and social support demonstrated significant negative correlations with depression (*r* = −0.433, *p* < 0.01; *r* = −0.434, *p* < 0.01).

**Table 2:** Results of the descriptive statistics and correlation analysis (*n* = 564).

Variable	Reliability	M ± SD	1	2	3	4
1. Physical exercise	0.623	31.38 ± 20.871	1			
2. Academic satisfaction	0.736 ~ 0.940	46.18 ± 10.280	0.222^**^	1		
3. Social support	0.965	67.74 ± 16.467	0.160^**^	0.683^**^	1	
4. Depression	0.794	42.94 ± 5.844	−0.133^**^	−0.433^**^	−0.434^**^	1

**p < 0.01.

### 4.3 Research hypothesis testing

**Figure 1:**
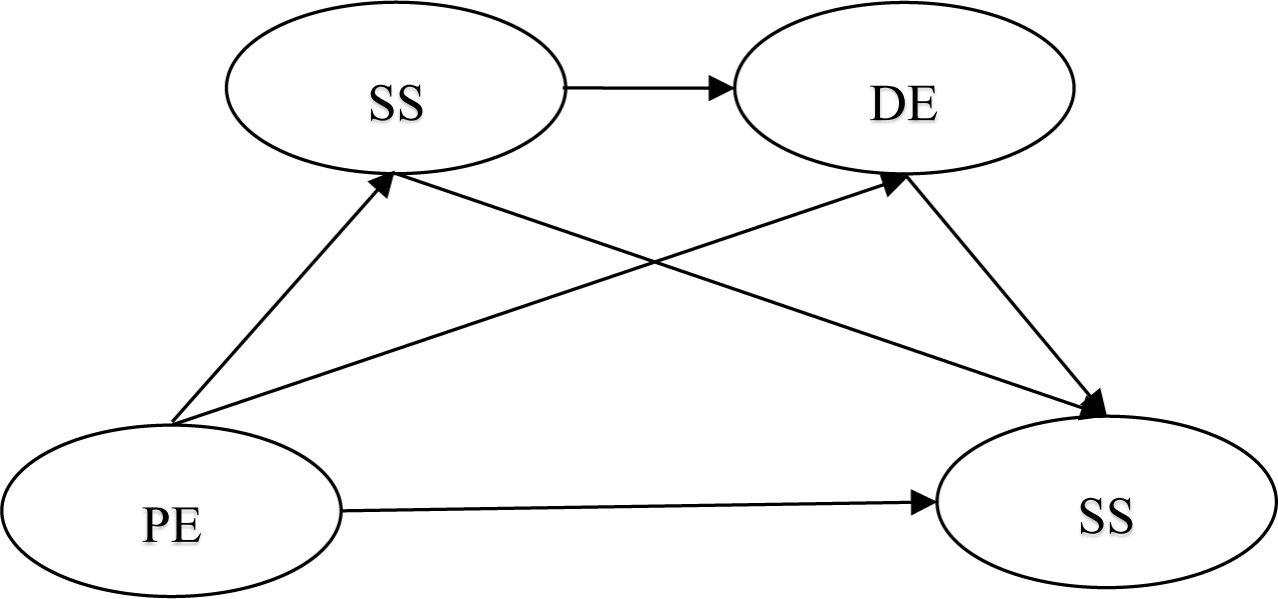
Mediation model from physical exercise to academic satisfaction. PE, physical exercise; SS, social support; AS, academic satisfaction; DE, depression.

The research hypotheses 1 to 8 were examined by using the PROCESS V4.1 in SPSS 27.0 software. Based on the findings from Table 3 and Figure 1, the standardized path coefficients for H1 and H2 are estimated at 0.119 (*t* = 3.833, *p* < 0.001) and 0.159 (*t* = 3.850, *p* < 0.001) respectively, indicating a significant positive impact of physical exercise on academic satisfaction and social support. Therefore, the data analysis results provide support for H1 and H2. The standardized path coefficient for H3 is found to be −0.132 (*t* = −3.192, *p* < 0.01), suggesting a significant negative impact of physical exercise on depression; thus supporting H3. Furthermore, social support positively influences academic satisfaction with an estimated path coefficient of 0.582 (*t* = 17.346, *p* < 0.001), confirming support for H4. Additionally, both H5 and H6 show that academic satisfaction has a negative effect on depression (*t* = −11.256, *p* < 0.001) and that depression also negatively affects academic satisfaction (*t* = −5.047, *p* < 0.00 L). These findings support both hypotheses H5 to H6.

**Table 3:** Standardization path coefficients and hypothesis testing results (*n* = 564).

Path	β	SE	T	*p*	Test result
H_1_: PE → AS	0.119	0.011	3.833	<0.001	Support
H_2_: PE → SS	0.159	0.024	3.850	<0.001	Support
H_3_: PE → DE	−0.132	0.007	−3.192	<0.01	Support
H_4_: SS → AS	0.582	0.021	17.346	<0.001	Support
H_5_: SS → DE	−0.428	0.011	−11.256	<0.001	Support
H_6_: DE → AS	−0.169	0.071	−5.047	<0.001	Support

PE, physical exercise; SS, social support; AS, academic satisfaction; DE, depression.

As presented in Table 4, the 95% confidence intervals of the total effect of physical exercise on academic satisfaction range from 0.053 to 0.112, excluding zero, indicating a significant total effect (*t* = 0.082). The indirect effect (i.e., mediating effect) is estimated to be between 0.024 and 0.063 at a 95% confidence interval, also excluding zero, suggesting the presence of a mediating effect between physical exercise and academic satisfaction. Furthermore, the direct effect is estimated to be between 0.017 and 0.061 at a 95% confidence interval without including zero, demonstrating that physical exercise has a significant direct impact on academic satisfaction. Therefore, it can be concluded that social support (0.035) and depression (0.004) partially mediate this relationship. Additionally, our study reveals evidence for chain mediation (0.004) through social support and depression in the relationship between physical exercise and academic satisfaction. Therefore, H7 and H8 are supported.

**Table 4:** The results of mediation analysis using bootstrapping (*n = 564*).

Path	Effect	SE	95%CI		Effective dose
			Lower	Upper	
Direct effect	0.039	0.111	0.017	0.061	47.50%
Indirect effect	0.043	0.010	0.024	0.063	52.38%
PE → SS → AS	0.035	0.010	0.020	0.059	42. 87%
PE → DE → AS	0.004	0.020	0.017	0.095	4.63%
PE → SS → DE → AS	0.004	0.002	0.002	0.007	4.87%
Total effect	0.082	0.015	0.053	0.112	

PE, physical exercise; SS, social support; AS, academic satisfaction; DE, depression; bootstrapping = 5,000 samples.

## 5 Discussion

**Figure 2:**
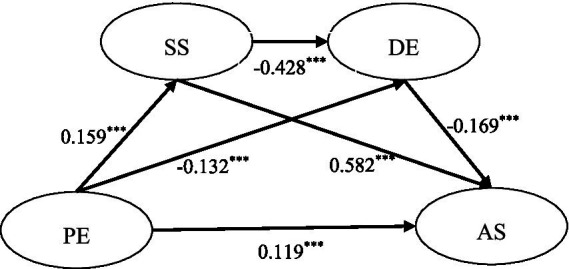
Mediation model from physical exercise to academic satisfaction. CFI = 0.932; GFI = 0.922; RMSEA = 0.04; ***p < 0.001, **p < 0.01; PE, physical exercise; SS, social support; AS, academic satisfaction; DE, depression.

The present study provides empirical evidence supporting the significant positive impact of physical exercise on academic satisfaction among middle school students, which is consistent with previous findings of Moral-Garcia et al. (2021) and Donnelly et al. (2016), and expands upon the results reported by Singh et al. (2012). Drawing from self-determination theory, it is argued that physical exercise plays a crucial role in enhancing individuals’ intrinsic motivation, thereby leading to increased levels of happiness and satisfaction (Teixeira et al., 2012). Meyer et al. (2021) further demonstrated that improvements in students’ intrinsic motivation through physical exercise significantly contribute to their overall life satisfaction, with academic satisfaction being identified as an important factor influencing life satisfaction (Balaguer et al., 2017). Additionally, research suggests a subtle relationship between students’ health and academic performance, which serves as a key predictor of academic satisfaction (Baños et al., 2019). Active engagement in sports activities has been shown to greatly enhance student health and improve academic performance, ultimately resulting in higher levels of academic satisfaction among middle school students (Asigbee et al., 2018). Furthermore, previous studies have confirmed that physical exercise not only alleviates academic pressure but also enhances interpersonal communication skills among students (Haugland et al., 2003; Rackow et al., 2014). Therefore, the findings of this research highlight the importance of active participation in physical activity for promoting greater levels of academic satisfaction among middle school students (Figure 2).

Building on previous studies’ findings (Babiss and Gangwisch, 2009a; Chen et al., 2021a), this study further examines the influence of physical exercise on academic satisfaction by using social support as a mediating variable. Consistent with the results of Chan et al. (2022), the research findings confirm the significant positive impact of social support on academic satisfaction. As a crucial personal resource (Feng et al., 2018), social support plays a vital role in maintaining good mental and emotional health and promoting physical and mental well-being (Chen et al., 2016). For adolescents, social support can enhance their motivation to learn, thereby boosting their academic satisfaction (Jones, 2008). Therefore, enhancing students’ social support can foster positive emotions that contribute to improved academic satisfaction (Jia and Cheng, 2024). Similarly, physical exercise significantly promotes social support among middle school students. Students have established robust networks of social support through communication and interaction with others during sport exercise (Martínez-Martí and Ruch, 2016), making them more likely to receive assistance from family and friends. In essence, physical activity has always been linked to social support (Mendonça et al., 2014). Thus, actively participating in physical exercise increases the likelihood of receiving help from family members, friends, and society while also improving learning motivation and academic performance for higher levels of academic satisfaction. Considering the current situation of Chinese education, which often emphasizes scores (e.g., score-only theory), parental support might be more inclined toward providing emotional comfort rather than guidance on learning.

In line with the findings of Foroughi et al. (2022) and Pekrun et al. (2002), this study confirms that depression has a significantly negative impact on academic satisfaction. Depression is a detrimental mental state that can impair overall functioning in individuals (Wagner et al., 2022). Depression may increase the risk of academic decline (McCurdy et al., 2023) for students, consequently leading to dissatisfaction with their academic performance. Previous study confirmed that higher levels of positive emotions among students were associated with improved academic performance and greater satisfaction (Schütz et al., 2013). Similarly, regular physical exercise significantly reduces the incidence of depression (Noetel et al., 2024). Individuals who engage in consistent physical activity exhibit enhanced self-confidence, values, life satisfaction, and reduced anxiety and depression levels (Esmaeilzadeh, 2015; Wu et al., 2016). To some extent, students who regularly participate in physical education are more likely to experience positive emotions, decreased anxiety and depression symptoms, as well as demonstrate better academic performance and increased academic satisfaction. Building upon previous researches by Dorfman (2015) and Kayani et al. (2018), this study contributes to understanding the influence of physical exercise on academic satisfaction by considering depression as a mediating variable.

Consistent with the findings of Mizuta et al. (2017), social support significantly predicts lower levels of depression, indicating that higher levels of social support are associated with reduced occurrence of negative emotions such as depression in adolescents. According to Maslow’s hierarchy of needs theory, unmet interpersonal communication needs can result in decreased social support and an increased likelihood of experiencing depression (Maslow, 1968). This highlights the importance of social support as a crucial psychological resource (Thoits, 2011) that positively influences anxiety, depression, and other adverse emotions, enabling individuals to effectively cope with challenges or setbacks while serving as a protective factor against depression.

Physical exercise exerts a combined influence on the academic satisfaction of middle school students via a chain mediating effect involving social support and depression. This finding aligns with Ruiz-Comellas et al. (2022), providing evidence for the importance and significance of social support and depression in relation to middle school students’ academic satisfaction. Social support obtained during physical exercise plays a crucial role in influencing depression among middle school students, with a stable correlation observed between social support and depression (Khan et al., 2020). In addition, physical exercise can enhance both internal and external sources of social support for middle school students, thereby fostering positive emotions development while mitigating depressive symptoms (Kayani et al., 2018). Consequently, engaging in physical exercise can enhance individuals’ capabilities and value (Jackson et al., 2022), facilitate greater access to social support networks (Lau et al., 2016), alleviate psychological pressure, reduce the risk of developing depression (Salmon, 2001), ultimately leading to improved academic performance and satisfaction levels (Trudeau and Shephard, 2008). Therefore, this finding provides a new perspective for exploring the potential mechanisms by which physical exercise affects academic satisfaction among middle school students. Furthermore, as suggested by Cohen (1988), the statistical significance of the chain mediation effect value (i.e., 0.004) in this study has certain limitations. However, we can make appropriate interventions to the chain-like intermediary mechanism, thereby enhancing the impact of physical exercise on academic satisfaction.

The following limitations and future perspectives are discussed based on the aforementioned findings. This study only chooses 5 schools for data collection in the Yuecheng District of Shaoxing City. Cross-regional sample method should be carried out in the future to make the research conclusions more representative. Secondly, demographic variables were used as control variables to examine all the research hypotheses. Future research should explore the moderating effects of these variables on the research hypotheses model. Lastly, this study confirmed the relationship between physical exercise and academic satisfaction by using social support and depression as mediating variables, which could not fully explain the effect of physical exercise on middle school students’ academic satisfaction. Additional mediating variables should be explored to clarify the role of physical exercise on academic satisfaction.

## 6 Conclusion

Utilizing middle school students as research sample, this study investigates the impact of physical exercise on academic satisfaction by constructing a structural equation model. The findings indicate that physical exercise significantly and positively influences both academic satisfaction and social support. Moreover, physical exercise and social support exhibit a negative correlation with depression, while depression has a significant negative effect on academic satisfaction. Furthermore, Social support and depression mediate the relationship between physical exercise and academic satisfaction. These results contribute to understanding the underlying mechanisms through which physical exercise affects academic satisfaction among middle school students.

From a theoretical perspective, the findings support the positive influence of physical exercise on academic satisfaction. These results also extend previous research (Kayani et al., 2018; Moksnes et al., 2016) by examining the mediating role of social support and depression. That is, they also contribute to explaining the influential mechanism of physical exercise on academic satisfaction. From a practical standpoint, managers should establish a school-family-community collaboration mechanism to provide students with the necessary social support and positive experiences during physical exercise. Teachers ought to incorporate team sports projects, such as basketball, into the school curriculum to simultaneously improve social support and intervene in depression. For middle school students, they should strive to establish good interpersonal relationships and ensure a flow experience during physical exercise.

## Data Availability

The original contributions presented in the study are included in the article/supplementary material, further inquiries can be directed to the corresponding author. Requests to access these datasets should be directed to thbzhy@163.com.
